# Optimization of drug–target affinity prediction methods through feature processing schemes

**DOI:** 10.1093/bioinformatics/btad615

**Published:** 2023-10-09

**Authors:** Xiaoqing Ru, Quan Zou, Chen Lin

**Affiliations:** Department of Computer Science, University of Tsukuba, Tsukuba, Japan; Institute of Fundamental and Frontier Sciences, University of Electronic Science and Technology of China, Chengdu, China; Yangtze Delta Region Institute (Quzhou), University of Electronic Science and Technology of China, Quzhou, Zhejiang, China; Department of Computer Science and Technology, School of Informatics, Xiamen University, Xiamen, Fujian, 361005, China

## Abstract

**Motivation:**

Numerous high-accuracy drug–target affinity (DTA) prediction models, whose performance is heavily reliant on the drug and target feature information, are developed at the expense of complexity and interpretability. Feature extraction and optimization constitute a critical step that significantly influences the enhancement of model performance, robustness, and interpretability. Many existing studies aim to comprehensively characterize drugs and targets by extracting features from multiple perspectives; however, this approach has drawbacks: (i) an abundance of redundant or noisy features; and (ii) the feature sets often suffer from high dimensionality.

**Results:**

In this study, to obtain a model with high accuracy and strong interpretability, we utilize various traditional and cutting-edge feature selection and dimensionality reduction techniques to process self-associated features and adjacent associated features. These optimized features are then fed into learning to rank to achieve efficient DTA prediction. Extensive experimental results on two commonly used datasets indicate that, among various feature optimization methods, the regression tree-based feature selection method is most beneficial for constructing models with good performance and strong robustness. Then, by utilizing Shapley Additive Explanations values and the incremental feature selection approach, we obtain that the high-quality feature subset consists of the top 150D features and the top 20D features have a breakthrough impact on the DTA prediction. In conclusion, our study thoroughly validates the importance of feature optimization in DTA prediction and serves as inspiration for constructing high-performance and high-interpretable models.

**Availability and implementation:**

https://github.com/RUXIAOQING964914140/FS_DTA.

## 1 Introduction

In drug–target interaction research, wet experiments have been complemented by computational methods due to time and resource constraints (Chen *et al.* 2016). A variety of computational concepts and methods have been applied to drug–target affinity (DTA) prediction and have achieved progressive results (Yamanishi *et al.* 2010, Chen *et al.* 2018, Huang *et al.* 2021, Chen *et al.* 2023a). Upon summarizing the characteristics of existing methods, we find that suboptimal performance can be attributed to two main factors: (i) the sparsity of samples, an inherent obstacle that cannot be immediately overcome or resolved in the short term within this research topic; and (ii) inappropriate design of various steps during the model construction. Consequently, most studies concentrate on aspects of design and model construction that can be improved through human intervention (Mei *et al.* 2013, Pahikkala *et al.* 2015, Luo *et al.* 2017, Wang and Zou 2021, Zeng *et al.* 2021, Ru *et al.* 2022, Yuan *et al.* 2022, Chen *et al.* 2023b).

The process of building a machine-learning model for DTA prediction can be divided into four steps: data collection and processing, feature extraction and optimization, learner selection, and model training and testing. Feature extraction and optimization is an important step that has a significant impact on improving model performance, enhancing robustness, and increasing interpretability. Many existing studies aim to comprehensively characterize drugs and targets by extracting features from multiple perspectives, but this approach has its drawbacks: (i) inevitable information overlap between differing perspectives may lead to an abundance of redundant or noisy features; and (ii) feature sets extracted from multiple perspectives tend to have high dimensionality, which requires more storage space and potentially leads to the curse of dimensionality or overfitting of the training data.

**Figure 1.**
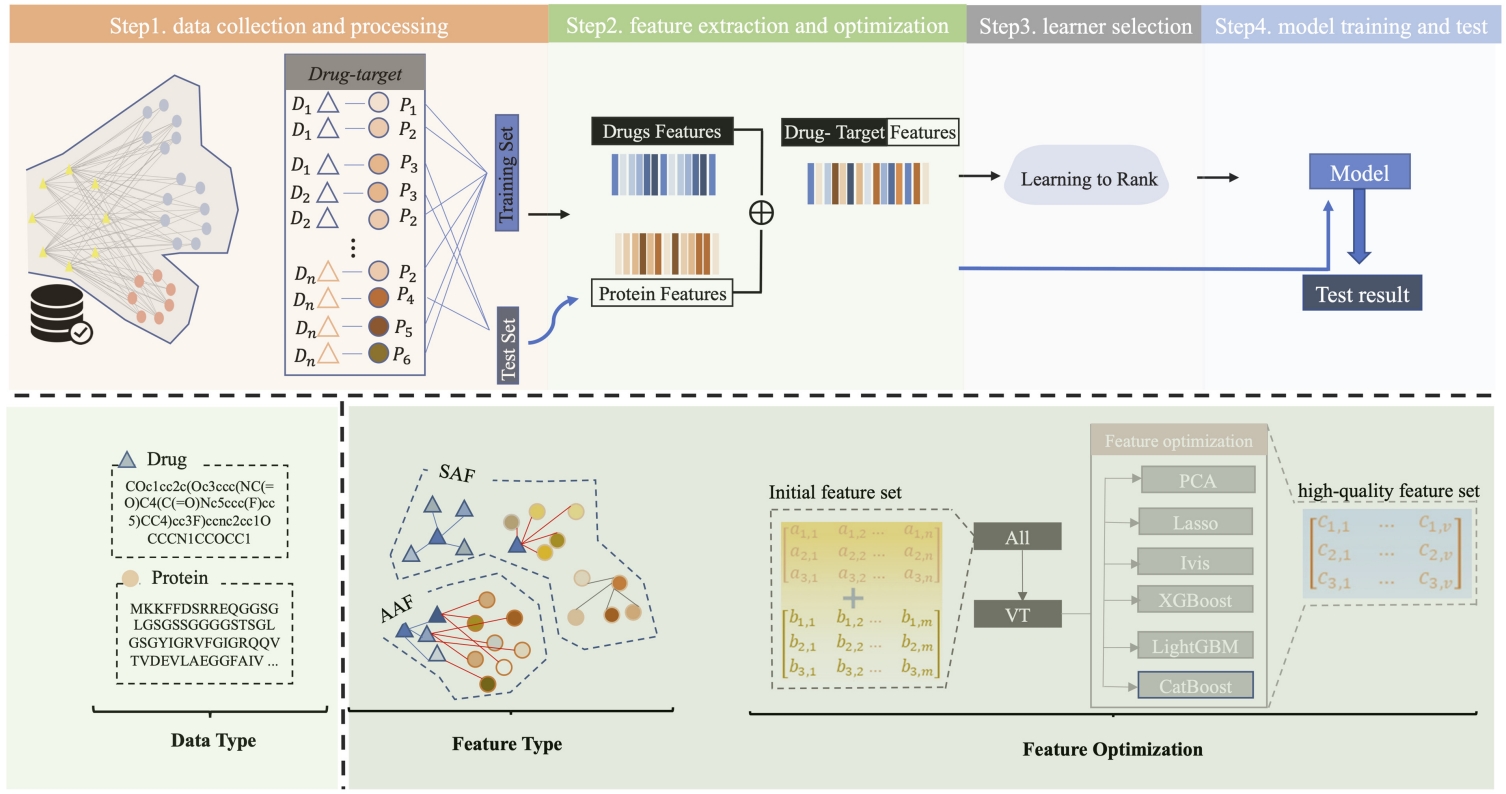
The overall architecture of this study.

In this study, we first extract self-associated features (SAFs) and adjacent-associated features (AAFs) of drugs and targets based on their similarity and sharing properties. Then, SAFs and AAFs are optimized by eliminating low-variance features and employing methods, such as Principal Component Analysis (PCA), Least Absolute Shrinkage and Selection Operator (Lasso), ivis, XGBoost, LightGBM, and Catboost. Subsequently, the optimized feature sets are input into a learning to rank (LTR) algorithm—multiple Additive Regression Tree (MART) for DTA prediction. The overall architecture of this study is illustrated in Fig. 1. Extensive experiments on two widely used datasets show that XGBoost, LightGBM, and Catboost are most effective methods for achieving good performance and strong robustness.

To enhance the model’s interpretability and inspire future research, we assess feature importance based on Shapley Additive Explanations (SHAP) values, and find that the top 150D features constitute the high-quality feature set and the top 20D features have a breakthrough impact on the DTA prediction for two datasets through the incremental feature selection (IFS) approach. Furthermore, we observe the common features appearing in the top 20 features ranked by XGBoost, LightGBM, and Catboost, and analyze the type of these features. It can be concluded that different types of features exhibit distinct performances under different circumstances, but both AAFs and SAFs positively influence model performance.

## 2 Materials and methods

### 2.1 Feature extraction

AAFs and SAFs are extracted according to the drug–drug similarity, drug–drug sharing, protein–protein similarity, protein–protein sharing, and drug–protein binding affinity information. Drug–drug similarity is the 2D chemical structure similarity calculated with the structure clustering server at PubChem. Protein–protein similarity is the sequence similarity represented by the normalized Smith–Waterman [16] value. Drug–drug sharing is the number of shared targets between two drugs. Protein–protein sharing is the number of shared drugs between proteins.

The representation and source of SAFs are as follows:


(1) $$\mathrm{SAF}=\left\{\partial_{i}⊕\beta_{i}⊕\gamma_{1}\right\}.$$

And,


(2) $$\left\{\partial_{1},\partial_{2},\partial_{3},\partial_{4},\partial_{5}|\partial_{i}\in f_{i}[S]\right\},$$

(3) $$\left\{\beta_{1},\beta_{2},\beta_{3},\beta_{4},\beta_{5},\beta_{6},\beta_{7-11},\beta_{12-16}|\beta_{i}\in f_{i}[A^{-}]\right\},$$

(4) γ1|γ1=∑n=1ma1nm, a1n∈A.

Where S represents the similarity matrix between drugs or between targets, A represents the affinity matrix between drugs and targets, $A^{-}$ represents the matrix obtained after removing unknown affinity elements from A. $f_{i}$ represents a function that obtains special values of a set. $\partial_{1}–\partial_{5}$ represent the mean, 50th percentile, 75th percentile, 85th percentile, and 95th percentile of the elements in the S, respectively. $\beta_{1}–\beta_{6}$ represent the mean, count, mode, 25th percentile, 50th percentile, and 75th percentile of the elements in the $A^{-}$, respectively. $\beta_{7-11}$ represent the higher five affinity values, respectively. $\beta_{12-16}$ represent the lower five affinity values, respectively.

AAFs are features of the target object that are derived from the SAFs of its neighbors. There are two ways to obtain neighbors: (i) drugs or targets that exhibit a level of similarity or sharing with the target object that exceeds a predefined threshold. (ii) The top 5 drugs or targets with the utmost similarity to the target object, as well as the top 5 drugs or targets most similar to each of these five drugs or targets.

### 2.2 Feature optimization

In this study, various feature selection and dimensionality reduction techniques are employed to optimize the initially obtained feature set, including commonly used technique like PCA, a deep learning-based tool like ivis, and several regression-based methods, such as LASSO, XGBoost, LightGBM, and Catboost. Notably, XGBoost, LightGBM, and Catboost are GBDT variants that adopt an underlying principle: a strong learner is formed by integrating multiple weak learners.

#### 2.2.1  GBDT

GBDT, a classic version of the gradient boosting algorithm, operates on iteratively enhancing predictive accuracy by integrating multiple decision trees. Each constituent tree is constructed in response to the residuals produced by its immediate predecessor, with the ultimate prediction being the cumulative sum of predictions made by all regression trees (Ye *et al.* 2009, Rao *et al.* 2019).

The CART algorithm is the base learner in GBDT. A regression CART tree T(x,θ) can be defined as follows (Rao *et al.* 2019, Liang *et al.* 2020)


(5) $$T\left(x,\theta \right)=\sum_{j=1}^{J}c_{j}I\;\left(x\in R_{j}\right),$$

where J represents the leaf node tree, $c_{j}$ denotes the predicted value output by the regression tree, I stands for the event function, and $R_{j}$ represents the set of predicted values of the leaf nodes.

Given $\{x_{i},y_{i}{\}}_{i=1}^{N}$ denotes the features $x_{i}$ and label $y_{i}$ of the samples, where N represents the number of samples. The calculation process of GBDT can be described as follows:

firstly, the initial tree $f_{1}(x)$, which is obtained by directly fitting the target value using the regression tree, is defined as follows (Rao *et al.* 2019, Liang *et al.* 2020):


(6) $$f_{1}\left(x\right)=\underset{c}{\mathrm{argmin}}\sum_{i=1}^{N}L\left(y_{i},c\right),$$

where L is the loss function, and c is the initial constant value.

Then, the second to m-th regression trees are obtained through iterative training. The training objective for each tree is computed during this process in the following manner (Rao *et al.* 2019, Liang *et al.* 2020):


(7) $$r_{mi}=y_{i}-f_{m-1}\left(x_{i}\right)=-[\frac{\partial L\left(y_{i},f\left(x_{i}\right)\right)}{\partial f\left(x_{i}\right)}{]}_{f\left(x\right)=f_{m-1}\left(x\right)}.$$

The regression tree at the m-th iteration is defined as follows (Rao *et al.* 2019, Liang *et al.* 2020):


(8) $$f_{m}\left(x\right)=f_{m-1}\left(x\right)+T_{m}\left(x,\theta_{m}\right)=f_{m-1}\left(x\right)-\sum_{j=1}^{J}c_{mj}I\;\left(x\in R_{mj}\right),$$

and Rao *et al.* (2019) and Liang *et al.* (2020),


(9) $$c_{mj}=\underset{c}{\mathrm{argmin}}\sum_{x\in R_{mj}}{\left(r_{mi}-T_{m}(x_{i},\theta_{m})\right)}^{2}.$$

Finally, the predicted result is the sum of these regression trees (Rao *et al.* 2019, Liang *et al.* 2020):


(10) $$F\left(x\right)=\sum_{m=1}^{M}f_{m}\left(x\right).$$

#### 2.2.2  XGBoost

XGBoost (Chen and Guestrin 2016) is an upgraded version of GBDT. It introduces several optimizations in algorithm implementation, such as feature parallelization, cache awareness, and approximate algorithms for splittable nodes, to accelerate training and improve accuracy. Moreover, XGBoost applies L1 and L2 regularization techniques along with penalties based on feature importance to mitigate the propensity toward overfitting.

The objective function within XGBoost can be formulated as (Al Daoud 2019, Liang *et al.* 2020):


(11) $$O=\sum_{i=1}^{N}L\left(y_{i},{\tilde{y}}_{i}\right)+\sum_{m=1}^{M}\Omega (f_{m}),$$

and Liang *et al.* (2020),


(12) $${\tilde{y}}_{i}=\sum_{m=1}^{M}f_{m}(x_{i})={\tilde{y}}_{i}^{m-1}+f_{M}\left(x_{i}\right),$$

(13) $$\sum_{m=1}^{M}\Omega (f_{m})=\sum_{j=1}^{M-1}\Omega \left(f_{j}\right)+\Omega \left(f_{m}\right),$$

where L is the loss function, ${\tilde{y}}_{i}$ represents the final predicted value of the i-th sample, and $\Omega (f_{m})$ represents the regularization term for m iterations.

The objective function, along with the regularization term, is desired to be as small as possible. To find the $f_{M}$ that minimizes the objective function, XGBoost performs Taylor expansion of the loss function at the point ${\tilde{y}}_{i}^{m-1}$. Consequently, the objective function is as follows (Al Daoud 2019, Liang *et al.* 2020):


(14) $$O=\sum_{i=1}^{N}\left[g_{i}f_{M}\left(x_{i}\right)+h_{i}f_{M}^{2}\left(x_{i}\right)\right]+\Omega \left(f_{M}\right)=\sum_{i=1}^{N}\left[g_{i}W_{q(x_{i})}+{\frac{1}{2}h}_{i}W_{q(x_{i})}^{2}\right]+\Omega (f_{M}),$$

(15) $$\Omega \left(f_{M}\right)=\gamma T+\frac{1}{2}\lambda \sum_{t=1}^{T}{\left(w_{t}\right)}^{2},$$

where $g_{i}$, $h_{i}$ are the first derivative and second derivative of the loss function, respectively. $W_{q(x_{i})}$ represents the function assigning sample $q(x_{i})$ in the tree to its corresponding leaf node. $f_{m}(x_{i})$ represents the prediction result of the m-th tree for the sample $x_{i}$.

#### 2.2.3 LightGBM

LightGBM is an additional GBDT variant that builds upon XGBoost and accomplishes three optimizations, as shown in the following formula (Ke *et al.* 2017, Al Daoud 2019, Liang *et al.* 2020):


(16) LightGBM=XGBoost+Histogram+GOSS+EFB.

Histogram, a bucketing algorithm, reduces the quantity of candidate split points to decrease computational complexity and memory consumption. Gradient-based One-Side Sampling (GOSS) optimizes the feature sampling process by selecting samples with a gradient absolute value below a certain threshold while preserving samples with large absolute values. Exclusive Feature Bundling (EFB) improves efficiency by re-encoding the values of certain features and bundling multiple mutually exclusive features into a new feature.

#### 2.2.4 CatBoost

CatBoost (Hancock and Khoshgoftaar 2020) is an emerging GBDT variant that utilizes symmetric tree regularization techniques to tackle overfitting problems arising from direct calculations in multiple dataset permutations. Moreover, CatBoost is specifically designed to adeptly manage categorical features within GBDT, it effectively eliminates the impact of low-frequency features and noise in categorical variables on decision tree generation by creatively considering a prior distribution term when calculating node gains (Prokhorenkova *et al.* 2018), as demonstrated in the following equation (Prokhorenkova *et al.* 2018, Al Daoud 2019):


(17) $${\hat{x}}_{k}^{i}=\frac{\sum_{j=1}^{p-1}\left[x_{\sigma_{j},k}=x_{\sigma_{p},k}\right]Y_{\sigma_{j}}+aP}{\sum_{j=1}^{p-1}\left[x_{\sigma_{j},k}=x_{\sigma_{p},k}\right]+a},$$

where $\sigma_{j}$ represents the *j*-th data point, ${\hat{x}}_{k}^{i}$ denotes the *k*-th discrete feature of the *i*-th row of data in the training set, *a* is a prior weight, and *p* is the prior distribution term, and [] represents an indicator function.

#### 2.2.5 Ivis

Ivis (Tian and Tao 2020) is a non-linear technique based on Siamese Neural Networks (SNN). SNN consists of three identical networks, with each network composed of three dense layers and an embedding layer. The embedding layer is configured to a size of two, aiming to project high-dimensional data into a 2D space. The weights of these layers are initialized using the LeCun normal distribution.

For the embedding layer, a linear activation function is used, and the weights are initialized using Glorot uniform distribution. To mitigate overfitting, each dense layer is accompanied by a dropout layer with a default dropout rate of 0.1. The scaled Exponential Linear Units activation function is applied to the dense layers, defined as follows (Klambauer *et al.* 2017):


(18) $$\mathrm{selu}\left(x\right)=\lambda \left\{\begin{array}{c} x,\;\mathrm{if}\;x>0\\ a\exp\left(x\right)-a,\;\mathrm{if}\;x\;\leq 0 \end{array}\right..$$

The triplet loss function is employed as the training loss function for the model. K-nearest neighbors (Zhang 2016) are used to generate data for the triplet loss function, with the tuning parameter *k* set at 100. During each iteration, a point from the dataset is chosen as the anchor. A positive point is randomly selected from the *k* closest neighbors around the anchor, and a negative point is randomly chosen outside of these neighbors. The objective of the triplet loss function is to minimize the distance between the anchor and the positive point while simultaneously maximizing the distance between the anchor and the negative point. The triplet loss function can be obtained as follows (Tian and Tao 2020):


(19) $$L_{\mathrm{tru}}\left(\theta \right)=\left[\sum_{a,p,n}D_{a,p}-\min(D_{a,n},D_{p,n})+m\right],$$

where a,p, and n represent the anchor, positive, and negative points, respectively, *D* signifies the Euclidean distance and *m* represents the margin.

### 2.3Feature importance calculation

SHAP (Lundberg and Lee 2017) is a method rooted in game theory designed to improve machine-learning model interpretability and performance assessment. SHAP assigns an importance value to each feature in relation to a specific prediction. Aggregating these SHAP values across all instances provides a global explanation of the model’s behavior.

For a given feature i, its corresponding Shapley value can be expressed as follows (Lundberg and Lee 2017):


(20) $$\emptyset_{i}=\sum \frac{\left|S|!(n-\left|S\right|-1\right)}{n!}\left[f\left(S\cup \left\{i\right\}\right)-f\left(S\right)\right],$$

where n represents the number of features, S represents the feature subset that does not include feature i, $\left|S\right|$ denotes the size of the feature subset, f(S) indicates the model prediction value with the feature subset S, and $f\left(S\cup \left\{i\right\}\right)$ represents the prediction value after adding feature i to the subset S.

### 2.4 Learning to rank

LTR (Cao *et al.* 2007, Liu 2009) strives to sort a set of objects based on relevance or priority. A typical application of this is when a user inputs a query into a search engine; a series of related documents are returned and presented in descending order of relevance to the query. In fact, the relationship between the documents and the query fed into LTR is one-to-many, as is the association between drugs and their targets. Therefore, LTR is applicable to DTA prediction. The multiple Additive Regression Tree (Burges *et al.* 2005) used in this study can be considered an LTR algorithm with regression properties, as it not only focuses on the relative order of objects but also emphasizes the fit between predicted values and actual values.

## 3 Experiments and results

### 3.1 Datasets and evaluation metrics

In this study, numerous experiments are conducted in two different scenarios across two commonly used datasets. The two scenarios include S1—predicting the association between known targets and new drugs with proteins as the queries, and S2—predicting the association between known drugs and new targets with drugs as the queries. We consider three evaluation metrics: concordance index (CI) (Gönen and Heller 2005), mean squared error (MSE), and $r_{m}^{2}$ (Öztürk *et al.* 2018, Nguyen *et al.* 2021).

The basic information of the datasets is shown in Table 1.

**Table 1.** The basic information of the datasets.^a^

Dataset	Protein/S1_qid	Drug/S2_qid	Training	Testing
Davis	442	68	25 046	5010
KIBA	229	2111	98 545	19 709

a S1_qid represents the number of queries in S1. S2_qid represents the number of queries in S2.

### 3.2 Parameter setting

In this study, we use grid search to determine the optimal parameters for each feature optimization method within specified ranges. Table 2 shows the hyperparameters and the specified search range involved in each method.

**Table 2.** The hyperparameters and the specified search range involved in each method.^a^

Method	Parameter setting
VarianceThreshold	Threshold: numpy.linspace (0.01,0.1,5)
PCA	n_components: mle, svd_solver: full
Lasso	alpha
XGBoost	n_estimators: [200,400,600,800], max_depth: [3,4,5,6], learning_rate: [0.02,0.04,0.06,0.08]
LightGBM	boosting_type: gbdt, n_estimators: [200,400,600,800], max_depth: [3,4,5,6], learning_rate: [0.02,0.04,0.06,0.08]
CatBoost	iterations: [200,400,600,800], depth: [3,4,5,6], loss_function: RMSE, eval_metric: RMSE, min_data_in_leaf: [2,3,4,5], learning_rate: [0.02,0.04,0.06,0.08]
Ivis	embedding_dims: [128, 256, 512,1024,2048], *k*: range(10,151,30), n_epochs_without_progress: range(10,21,5), model: maaten, supervision_metric: mse

a range(start, stop[, step]) is a function that represents a sequence of numbers starting from “start” and ending at “stop” with a step size of “step.” numpy.linspace (start, stop, and num) represents a function that returns “num” evenly spaced numbers between “start” and “stop.”

Variance threshold (Fida *et al.* 2021) is employed to eliminate features with low variance. It operates by calculating the variance of each feature and subsequently removing features with variance below a predetermined threshold. The “threshold” is a user-defined parameter that dictates the criteria for retaining or removing features.

PCA (Wold *et al.* 1987) is a widely used unsupervised linear dimensionality reduction method, designed to decrease feature dimensionality while preserving the maximum variance among the features. “n_components” is the main parameter used to determine the number of features, with “mle” signifying the automatic determination of feature dimensions based on the input data. “svd_solver” represents the approach for solving singular value decomposition (SVD), with “full” indicating the employment of the standard SVD method.

Lasso is a regularization method for linear regression that facilitates feature selection and sparse representation of model parameters by adding an L1 regularization term to the loss function (Tibshirani 1996). The primary parameter, “alpha,” signifies the weight of the L1 regularization term. In this study, an appropriate alpha value is determined through 5-fold cross-validation.

For XGBoost (https://xgboost.readthedocs.io/en/stable/), “learning_rate” controls the contribution of each iteration (i.e. each tree) to the final prediction result. “n_estimators” represents the number of trees, while “max_depth” denotes the maximum depth for each tree.

For LightGBM (https://lightgbm.readthedocs.io/en/latest/index.html), “boosting_type” refers to the boosting type, with “gbdt” being the default selection for this study. The meanings of the other three parameters are consistent with those in XGBoost.

For CatBoost (https://catboost.ai/en/docs/), it is imperative to establish the loss function, with this study electing to employ the Root Mean Square Error (RMSE). “iterations” refers to the number of trees to be constructed. “eval_metric” serves to delineate the performance metric during the training procedure. “min_data_in_leaf” regulates the minimum number of samples in each leaf node of the decision tree, helping to prevent overfitting by ensuring the decision tree refrains from formulating leaf nodes that comprise an insufficient number of samples.

For Ivis (https://bering-ivis.readthedocs.io/en/latest/index.html), “n_epochs_without_progress” refers to the number of consecutive epochs without progress before training is terminated in the early stopping strategy. “k” represents the number of neighbors and affects the preservation of local structure in the low-dimensional space. Smaller values of “k” highlight the local structure, while larger values emphasize the global structure. “model” dictates the neural network architecture. “embedding_dims” is the dimension of the processed data. “supervision_metric” determines how to measure the difference between the processed data and the target (label) after dimensionality reduction.

### 3.3 Performance of various features

We conduct comparative experiments using SAFs and AAFs against two representative types of features to demonstrate their superiority. The methods involved are detailed as follows:

Gen: models that rely on features extracted from drug molecular descriptors (Cano *et al.* 2017) and basic protein sequence information (Bonidia *et al.* 2022).

Graph: models that utilize features obtained via the feature extraction algorithm in GraphDTA (Nguyen *et al.* 2021).

It can be observed from Table 3 that our method achieves excellent performance in both scenarios across the two datasets. Regarding accuracy and robustness, our features outperform both the Gen features, which are based on fundamental sequence information, and the Graph features that are learned by neural networks. Such a result implies that AAFs and SAFs are more effective in capturing drug and target information.

**Table 3.** Performance of various features.

	Davis						KIBA					
	S1			S2			S1			S2		
	CI	MSE	$r_{m}^{2}$	CI	MSE	$r_{m}^{2}$	CI	MSE	$r_{m}^{2}$	CI	MSE	$r_{m}^{2}$
Gen	0.886	0.283	0.654	0.875	0.308	0.616	0.839	0.209	0.699	0.836	0.213	0.686
Graph				0.885	0.263	0.672				0.870	0.163	0.760
Our method	0.937	0.149	0.818	0.931	0.162	0.798	0.895	0.130	0.813	0.893	0.131	0.807

### 3.4 Performance of various feature processing methods

The performance of various feature processing methods on two scenarios for two datasets are shown in Tables 4–7. “All” represents models built on all features. “VT” represents models built on the feature set after removing low-variance features.

**Table 4.** Performance of various feature processing methods for Davis.

	Davis (S1)				Davis (S2)			
	CI	MSE	$r_{m}^{2}$	num	CI	MSE	$r_{m}^{2}$	num
All	0.937	0.149	0.818	3208	0.931	0.162	0.798	3208
VT	0.937	0.151	0.816	3154	0.930	0.161	0.799	3154
PCA	0.915	0.182	0.777	620	0.903	0.204	0.746	620
Lasso	0.936	0.150	0.817	293	0.930	0.166	0.793	215
Ivis	0.877	0.307	0.625	128	0.856	0.348	0.566	128

**Table 5.** Performance of various feature processing methods for KIBA.

	KIBA (S1)				KIBA (S2)			
	CI	MSE	$r_{m}^{2}$	num	CI	MSE	$r_{m}^{2}$	num
All	0.895	0.130	0.813	3177	0.893	0.131	0.807	3177
VT	0.891	0.133	0.809	2625	0.890	0.135	0.801	2625
PCA	0.827	0.260	0.626	1367	0.822	0.261	0.617	1367
Lasso	0.891	0.134	0.806	557	0.888	0.138	0.797	325
ivis	0.754	0.420	0.395	128	0.748	0.429	0.369	128

**Table 6.** Performance of regression tree-based feature selection methods for Davis.

	Davis (S1)					Davis (S2)			
		CI	MSE	$r_{m}^{2}$	num	CI	MSE	$r_{m}^{2}$	num
XGB	SFM	0.938	0.150	0.816	1401	0.931	0.161	0.799	2235
	SHAP	0.938	0.150	0.816	1401	0.931	0.161	0.799	2235
Light	SFM	0.937	0.148	0.819	2108	0.931	0.161	0.800	2212
	SHAP	0.937	0.148	0.819	2108	0.931	0.161	0.800	2212
Cat	SFM	0.937	0.150	0.816	1278	0.930	0.162	0.798	1351
	SHAP	0.937	0.150	0.816	1278	0.930	0.162	0.798	1351

**Table 7.** Performance of regression tree-based feature selection methods for KIBA.

	KIBA (S1)					KIBA (S2)			
		CI	MSE	$r_{m}^{2}$	num	CI	MSE	$r_{m}^{2}$	num
XGB	SFM	0.891	0.133	0.809	2044	0.890	0.135	0.801	2256
	SHAP	0.891	0.133	0.809	2044	0.890	0.135	0.801	2256
Light	SFM	0.891	0.132	0.810	2127	0.890	0.135	0.801	1935
	SHAP	0.891	0.132	0.809	2127	0.890	0.135	0.801	1935
Cat	SFM	0.891	0.133	0.809	1105	0.890	0.135	0.802	1163
	SHAP	0.891	0.133	0.809	1105	0.890	0.135	0.802	1163

It can be observed from Tables 4 and 5 that low-variance features have a minimal impact on model performance. For Davis dataset, on S1, the model based on all features yields CI, MSE, and $r_{m}^{2}$ values of 0.937, 0.149, and 0.818. On S2, these values are 0.931, 0.162, and 0.798. After removing 54 low-variance features, the model performance decreases by ∼0.1%, which can be considered negligible. For KIBA dataset, which contains 552 low-variance features. On S1, the model based on VT features shows CI, MSE, and $r_{m}^{2}$ values of 0.891, 0.133, and 0.809. On S2, these values are 0.890, 0.135, and 0.801. The model performance in both scenarios is slightly inferior to that based on all features. However, prioritizing computational speed over model accuracy in the trade-off between the two is evidently a sensible approach.

Other feature processing methods optimize the feature set based on VT. As evidenced in Tables 4 and 5, the performance of the Ivis-based models is notably suboptimal, exhibiting limited robustness. This is particularly evident in both scenarios for KIBA dataset, where the model’s CI value is only around 75%, and $r_{m}^{2}$ falls below 0.5. The performance of the PCA-based model is significantly inferior to those built based on all features. Feature optimization techniques that rely on regression concepts exhibit superior performance. The LASSO-based model, which only uses 293D features can achieve CI and MSE values almost equivalent to those built on all features. It can be concluded from Tables 6 and 7 that the models utilizing the three feature optimization methods based on regression trees yield promising results, showing superior performance with low dimensions. Additionally, the robustness of these models is evidenced by $r_{m}^{2}$ values.

### 3.5 Performance of important features

As observed in Tables 4–7, the three feature selection methods—XGBoost, LightGBM, and CatBoost—all based on regression trees, show distinct advantages. To enhance the interpretability of the models, this study conducts a comprehensive analysis of the features selected by these three methods.

In addition to calculating the feature importance using the methods embedded in these three methods, this study ranks features according to their SHAP values. It is revealed that in Tables 6 and 7, the two feature importance calculation methods produce almost identical results, as they construct models with equivalent performance based on optimal feature subsets with the same dimensions.

**Figure 2.**
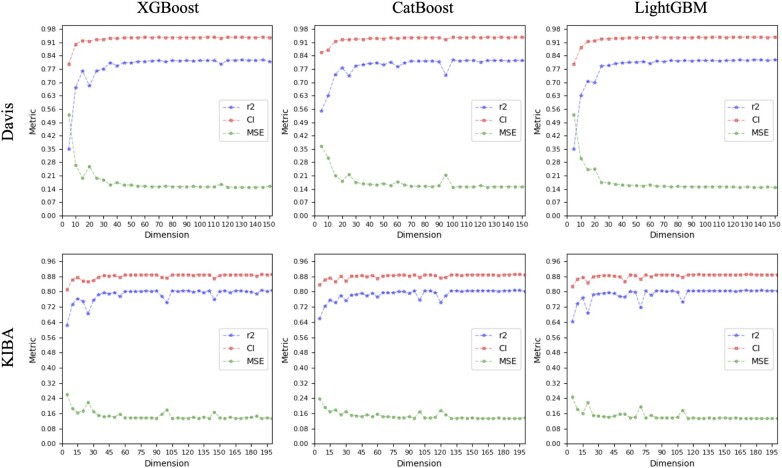
Performance of the model under each feature dimension on S1.

**Figure 3.**
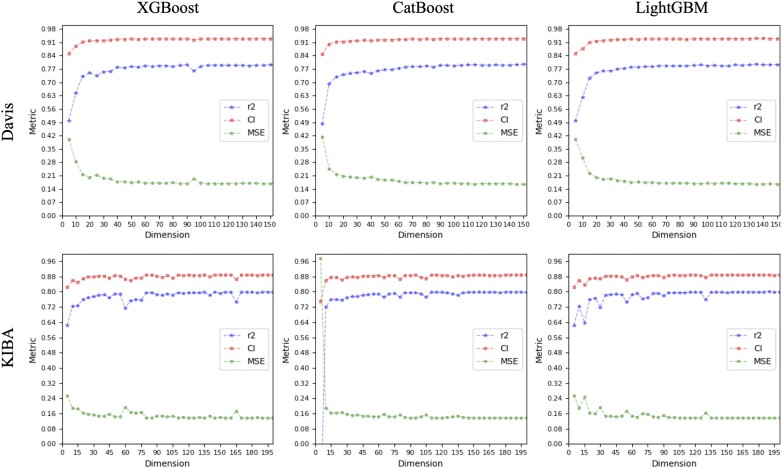
Performance of the model under each feature dimension on S2.

In this study, we obtain the optimal feature set by examining their SHAP values and using the IFS. Figures 2 and 3 illustrate the feature increment process with a step size of five on both scenarios for the two datasets, it is evident that the models’ performances tend to be stable when the feature dimension reaches 150. In other words, models built on the top 150 features perform almost as well as those built on all features, yet their dimensions constitute only one-twentieth of the latter. On both scenarios for KIBA dataset, the models using the three methods have CI values of ∼88.7%, MSE values below 14%, and $r_{m}^{2}$ around 79%. On both scenarios for Davis dataset, the CI, MSE, and $r_{m}^{2}$ values for the three methods are roughly 93%, 16%, and 80%, respectively.

### 3.6 Analysis of important features

**Figure 4.**
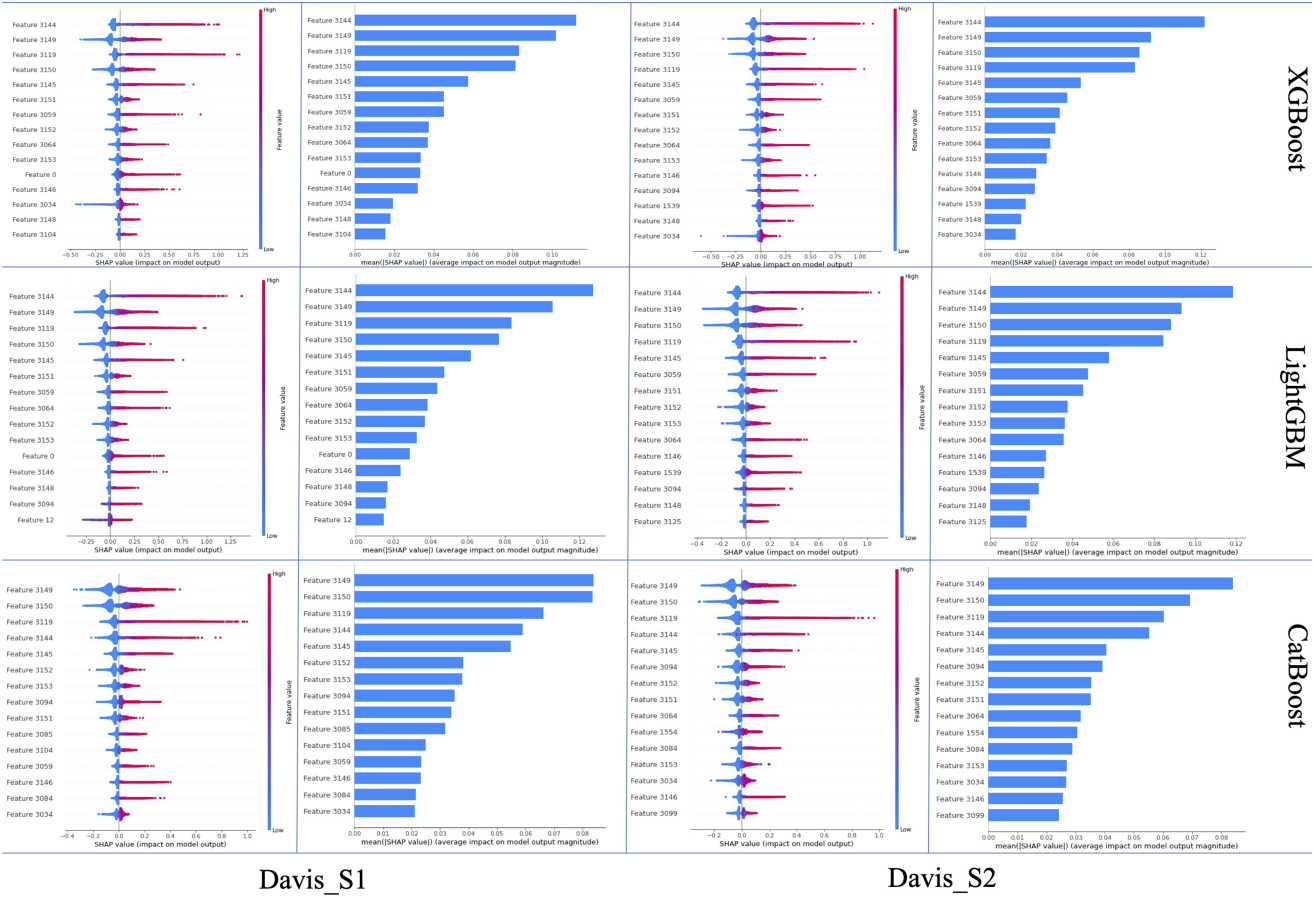
The top 20 features of regression tree-based feature selection methods for Davis.

**Figure 5.**
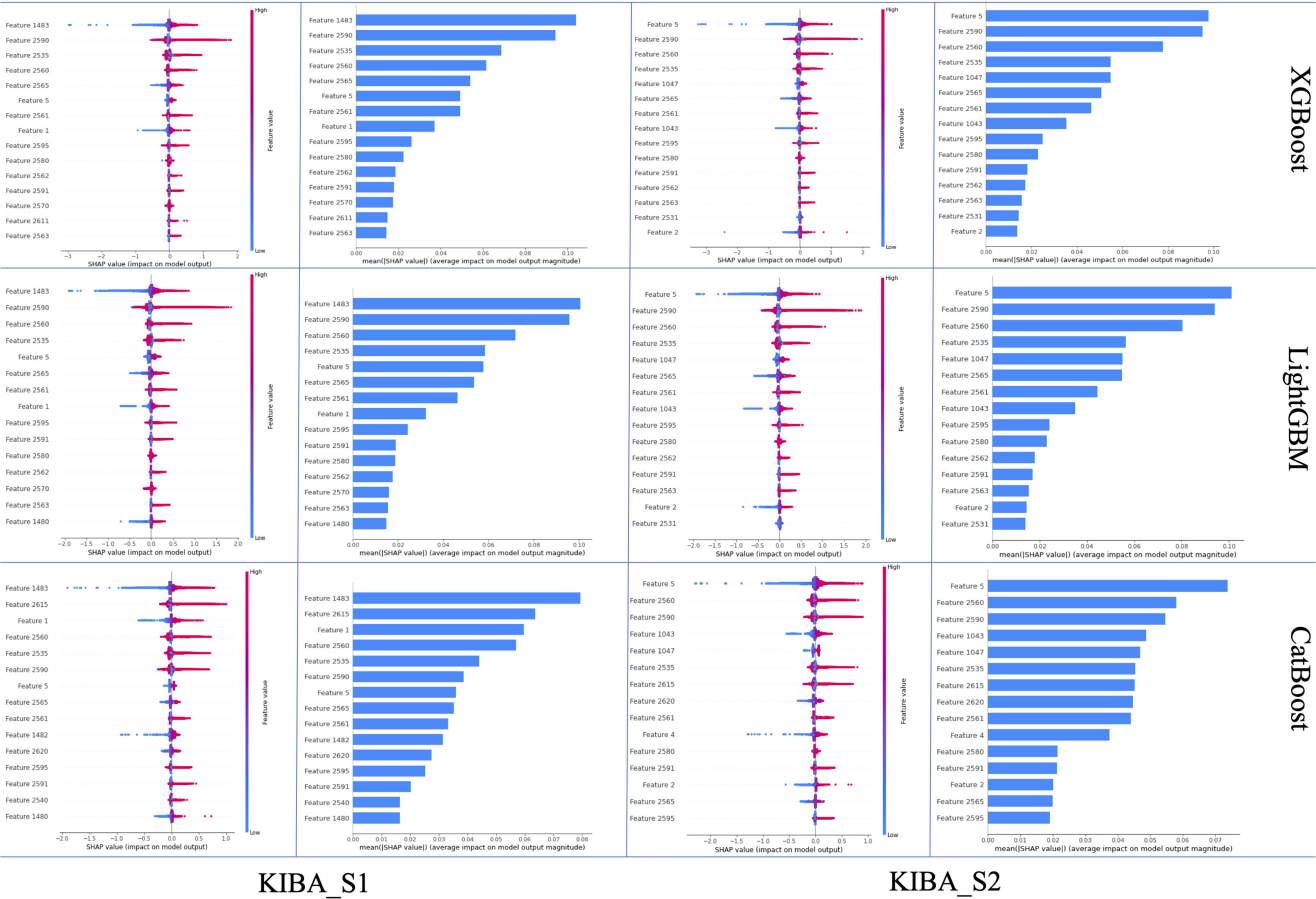
The top 20 features of regression tree-based feature selection methods for KIBA.

As depicted in Figs 2 and 3, the first 20 features significantly impact model performance. The top 20 features ranked by the three feature optimization methods are displayed in Figs 4 and 5.

For Davis, 15 common features appear among the top 20 ranked features in both scenarios S1 and S2, as determined by the three methods. Of these 15 features, 14 are AAFs—with 7 AAFs based on similarity and 7 AAFs based on sharing. The remaining feature is a SAF, which is derived from the DTA information of the target itself.

For KIBA, on S1, 14 common features among the top 20 ranked features are shared by the three regression algorithms. On S2, they share 15 common features. It is evident that similarity-based AAFs play a crucial role in both scenarios. On S1, 9 of the top 14 important features are AAFs, 8 of which are similarity-based. On S2, 10 of the top 15 important features are AAFs, 7 of which are similarity-based. Additionally, it is noteworthy that both scenarios share five SAFs derived from DTA information. This suggests that SAFs also demonstrate competitiveness within the KIBA dataset.

As a result, it can be concluded that different types of features exhibit varying performance under distinct circumstances. Although SAFs may not exhibit comparable efficiency as AAFs, it is undeniable that they share a complementary relationship, and each contributes positively to the overall performance of the model.

Furthermore, we can also observe the following: (i) some SAFs, such as the mean and quantile values of similarity between proteins/drugs, have little or even no impact on model performance. (ii) During SAFs extraction, overlapping neighbors for objects are obtained based on a set of similarity and sharing thresholds, which result in repetitive or redundant proximity information. (iii) When identifying additional neighbors that are most similar to the top 5 most similar neighbors, overlapping and intersecting information also occur. In summary, it is inevitable that the initially acquired features contain redundant and duplicate information. Consequently, feature optimization is a necessary process to build models with fast calculation speed, strong generalization ability, and high interpretability while maintaining consistent performance.

## 4 Conclusion

In this study, our main contribution lies in applying various feature processing techniques to optimize the extracted AAFs and SAFs, and then build a DTA prediction model with high accuracy and robust interpretability.

Extensive experimental results indicate that the three GBDT variant methods—Xgboost, LightGBM, and CatBoost—all exhibit superior feature selection capabilities. Furthermore, we employ SHAP values and IFS to rank significant features and determine the optimal feature subset. Models built on the optimal feature subset perform almost as well as those built on all features. In conclusion, feature optimization is crucial for building models that offer strong generalization ability and high interpretability while maintaining consistent performance.

There are still several avenues for enhancing DTA prediction methods, particularly with regard to feature engineering. For instance, one could leverage additional relationships to obtain more comprehensive feature information about drugs or targets from various perspectives, such as through protein–protein interactions or drug–disease associations. Additionally, most existing studies simply concatenate the features of drugs and targets. This approach may fail to fully explore and reflect deeper, underlying information and could lead to the curse of dimensionality. Therefore, deriving features from a range of perspectives and developing advanced feature selection or feature fusion methods will be central to our future endeavors.
